# *PLOS NTDs* celebrates our 10th anniversary: Looking forward to the next decade

**DOI:** 10.1371/journal.pntd.0006176

**Published:** 2018-01-25

**Authors:** Serap Aksoy, Judd L. Walson

**Affiliations:** 1 Department of Epidemiology of Microbial Diseases, Yale School of Public Health, New Haven, Connecticut, United States of America; 2 Departments of Global Health, Medicine (Infectious Disease), Pediatrics and Epidemiology, University of Washington, Seattle, Washington, United States of America

*PLOS Neglected Tropical Diseases* (*PLOS NTDs*) has celebrated its 10th anniversary! Under the exceptional leadership and guidance of Peter Hotez, Founding Editor, the journal was established in 2007 as a platform for research and advocacy for NTDs, a group of chronic endemic infections that largely affect poor populations in often unseen areas of the world—forgotten diseases of forgotten people [1,2]. Since 2007, there has been tremendous change in the field of NTDs, with a renewed interest in and commitment to reducing the impact of these devastating diseases affecting billions of individuals living in the most marginalized communities. In addition to the seven NTDs originally targeted by preventive chemotherapy (three soil-transmitted helminth infections, schistosomiasis, lymphatic filariasis [LF], onchocerciasis, and trachoma), visceral leishmaniasis and food-borne trematode infections have been recognized as priority diseases. Viral diseases, including dengue and rabies, have also emerged as important public health challenges in many of these populations [3]. While often considered diseases of resource-limited countries, the "blue marble health" concept has also emerged, recognizing the paradoxical NTD disease burden among the poor living in Group of Twenty (G20) nations and other wealthy countries, further supporting the need for these nations to take greater ownership in disease control as well as research and development [4]. Increased attention among policy makers, funders, and the public to the importance of these infections has attracted new scientific and financial resources towards research and product development for these poverty-promoting diseases.

The Public Library of Science, through *PLOS NTDs* and our sister journals, is proud to have played a small part in raising awareness, supporting advocacy, and assisting the community in creating a repository of high quality evidence and information to enable maximum impact of discoveries to combat these diseases. The signing of the London Declaration in 2012 and the subsequent WHO Roadmap for Implementation were milestones in efforts to define ambitious goals for NTD control, elimination, and eradication [5]. We recently reviewed the advances made towards the control of NTDs and noted that significant global progress has been made towards the elimination of many of these diseases, including African trypanosomiasis, dracunculiasis, LF, trachoma, and yaws [6]. However, while control efforts for other NTDs have expanded, less progress is evident for many of the other target diseases affecting public health, and new problems are emerging. We look forward to continuing our efforts in supporting evidence-based responses to these challenges in the coming decade.

We have been driven by dual goals since the launch of *PLOS NTDs*: to publish and present the most rigorous, well-conducted research within a strong open-access platform while also supporting science that originates among researchers and practitioners working in countries in which NTDs are endemic. As a community journal run by and for the NTD community, we remain committed to the mission and priorities at *PLOS NTDs* and are focused on improving the editorial process. We seek to actively support authors from disease-endemic countries to ensure that we publish the most relevant research in this field. We are also committed to serving as a resource for the publication of Viewpoint and Opinion pieces intended to identify gaps and suggest solutions where research is limited or lacking. As we move into the next decade, we are excited to launch new initiatives, such as a renewed Review section, in which *PLOS NTDs* will highlight the implications of cutting-edge research discoveries for control and policy decisions through commissioned articles.

In looking back over the past decade, we also a need to clarify the scope of the journal to ensure that our community is clear about the content that will remain in focus for *PLOS NTDs* and to ensure that we identify adequate content expertise to review and respond to submissions to the journal. When *PLOS NTDs* was launched, the list of “in-scope” diseases was relatively limited. However, over the past decade, the number of diseases and syndromes affecting marginalized individuals in resource-limited settings has continued to expand, ballooning the scope of the journal and occasionally leading to confusion for our authors and reviewers. Working closely with the NTD community, we will continue to publish research that is of direct human public health importance while working to refine and focus the scope of the journal to best meet the needs of our community.

While *PLOS NTDs* has benefited tremendously from the depth and breadth of research submitted to the journal, we recognize the need for a more consistent and rigorous approach to the review process. We are establishing systems to improve the methodologic rigor of published manuscripts in *PLOS NTDs*, including checklists for reviewers and the inclusion of strong epidemiologic and statistical experts on the review board. We are also working hard to improve our peer review time and encouraging more rapid decision-making through a variety of initiatives, including adapting the post acceptance publication process and facilitating preprint server submissions. Finally, we are actively working to improve the transparency of the review process, including the metrics by which article submissions are evaluated.

The next decade also offers the opportunity to more intensely focus on the inclusion of a diverse community in the operation of *PLOS NTDs*. We are committed to supporting the involvement of young investigators on our Editorial Board, particularly those from endemic countries and women scientists. Many of our Editorial Board members have participated in *PLOS NTDs* writing workshops around the globe to disseminate information on best-writing principles and guidelines, an initiative that we will continue. To successfully meet the ambitious targets set for NTD control, elimination, and eradication, we see the need to identify promising scientists early in their careers, including doctoral students, medical students, and postdoctoral trainees, and to expand the community of scientists working on these diseases by welcoming them into the PLOS community and providing them opportunity to contribute.

Unprecedented technological advancements have also led to the accumulation of vast amounts of demographic, geospatial, genetic, and genomic data on the agents of NTDs and their vectors [7–10]. As scientists are busy mining these data, the ground is set for novel translational discoveries that offer hope for transformative impact. *PLOS NTDs* is excited to continue our role in disseminating evidence, from basic science through translation and implementation science at scale, and we look forward to continuing to serve as a resource for the NTD community.

Now more than ever, there is a convergence of public interest, availability of funding, and improved evidence to support decision-making to address the NTDs. However, the landscape is also threatened by renewed attacks on science and the importance of evidence-based decision-making as political shifts threaten to undermine many of the gains made in recent years. As we enter the next decade, we look to ensure that the research and opinions published in *PLOS NTDs* are relevant to programs and policy makers and play a pivotal role in driving action to reduce the burden of these infections.

As a community journal run by working scientists, our priorities at *PLOS NTDs* for the coming decade remain the same—to publish and present the most rigorous research while also supporting science that originates among researchers and practitioners working in countries in which NTDs are endemic. We welcome your thoughts, your support, and your involvement.
